# All-trans retinoic acids induce differentiation and sensitize a radioresistant breast cancer cells to chemotherapy

**DOI:** 10.1186/s12906-016-1088-y

**Published:** 2016-03-31

**Authors:** Yunwen Yan, Zhen Li, Xiang Xu, Clark Chen, Wei Wei, Ming Fan, Xufeng Chen, Jian Jian Li, Yuan Wang, Jiaoti Huang

**Affiliations:** Institute of Clinical Pharmacology, Anhui Medical University, Hefei, China; Department of Biochemistry, Laboratory of Molecular Biology, Anhui Medical University, Hefei, China; Department of Pathology and Laboratory Medicine, University of California, Los Angeles, USA; School of Life Sciences, Anhui University, Hefei, China; Department of Radiation Oncology, University of California, Davis, USA

**Keywords:** Breast cancer, Radiation resistance, Cancer stem cell, ATRA

## Abstract

**Background:**

Radiotherapy is of critical importance in the treatment of breast cancer. However, not all patients derive therapeutic benefit and some breast cancers are resistant to the treatment, and are thus evidenced with prospective distant metastatic spread and local recurrence. In this study, we investigated the potential therapeutic effects of all-trans retinoic acid (ATRA) on radiation-resistant breast cancer cells and the associated invasiveness.

**Methods:**

The MCF7/C6 cells with gained radiation resistance after a long term treatment with fractionated ionizing radiation were derived from human breast cancer MCF7 cell line, and are enriched with cells expressing putative breast cancer stem cell biomarker CD44^+^/CD24^-/low^/ALDH^+^. The enhanced invasiveness and the acquired resistances to chemotherapeutic treatments of MCF7/C6 cells were measured, and potential effects of all-trans retinoic acid (ATRA) on the induction of differentiation, invasion and migration, and on the sensitivities to chemotherapies in MCF7/C6 cells were investigated.

**Results:**

MCF7/C6 cells are with enrichment of cancer stem-cell like cells with positive staining of CD44^+^/CD24^-/low^, OCT3/4 and NANOG. MCF7/C6 cells showed an increased tumoregensis potential and enhanced aggressiveness of invasion and migration. Treatment with ATRA induces the differentiation in MCF7/C6 cells, resulting in reduced invasiveness and migration, and increased sensitivity to Epirubincin treatment.

**Conclusion:**

Our study suggests a potential clinic impact for ATRA as a chemotherapeutic agent for treatment of therapy-resistant breast cancer especially for the metastatic lesions. The study also provides a rationale for ATRA as a sensitizer of Epirubincin, a first-line treatment option for breast cancer patients.

**Electronic supplementary material:**

The online version of this article (doi:10.1186/s12906-016-1088-y) contains supplementary material, which is available to authorized users.

## Background

Breast cancer is the leading cancer diagnosed in women and is second only to lung cancer in terms of cancer death, causing extensive morbidity and psychological distress to millions globally [1, 2]. Despite the tremendous efforts and progress in breast cancer research and early diagnosis, clinical outcome for breast cancer patients is still disappointing. Resistances to current therapeutic regimen, and as much as 40 % of relapses with recurrent and/or metastatic disease remain to be great challenges in clinical management for breast cancer patients [3–5]. It is also needed to be indicated that, while overall breast cancer mortality rates have decreased over the last several decades [6], the survival rates for metastatic breast cancer are currently estimated at less than 25 % for 5-year and 5–10 % for 10-year [3, 7–9].

Radiation therapy continues to be an important part of conditioning regimens for breast cancer treatment. Radiation therapy given after surgery in early stage breast cancer patients has shown significant effects of increasing the probability of both local control and survival [10]. The most recent meta-analysis including 10,801 women in 17 clinical trials of radiation or no radiation after lumpectomy showed that radiation reduced the 10-year risk of any recurrence in lymph node-negative women from 31 to 15.6 % and reduced the 15-year risk of death from breast cancer from 20.5 to 17.2 % [11]. However, the rate of totally control of tumor growth by radiotherapy remains unacceptable low, and studies have indicated that breast cancer patients may fail to radiation therapy and cancer cells in these patients become resistant to the treatment [12–15]. Elucidation of mechanism causing tumor radioresistance and definition of effective therapeutic targets to enhance tumor response, especially for the most resistant and aggressive cancer cells in the recurrent and metastatic lesions, are thus urgently needed.

In our previous studies, we observed a breast cancer MCF7 cell population (MCF + FIR) that could survive after a course of clinical fractionated doses of radiation and showed enhanced radioresistance compared to the wild type parental MCF7 cells [16, 17]. With sub-cloning, different clones with varied radiosensitivity were isolated from this radioresistant population [18]. Cells expressing the biomarkers of breast cancer stem cells (BCSCs; e,g., CD44^+^/CD24^-/low^/ALDH^+^) were further sorted and confirmed in one of these clones (MCF7/C6) [19], indicating that BCSCs can survive long-term fractionated radiation and be responsible tumor repopulation with radiation resistance. In supporting this observation, other studies also demonstrated the enrichment of cancer stem cells during the course of fractionated radiation [20, 21]. In addition, radiation is also shown to be able to reprogram the differentiated breast cancer cells into induced breast cancer stem cells (BCSCs) [22]. These and other results provide the evidence indicating that, while some patients with early-stage breast cancer can benefit from radiation therapy, others may gain resistance to radiotherapy with a potential of increased recurrence and/or distant metastasis due to the enrichment of BCSCs [23]. Thus, targeting BCSCs in patients with radiation resistant breast cancer may impede an important clinical impact for decreasing cancer metastatic potential in these patients.

In this study, we used the MCF + FIR cellular model to investigate the roles of BCSCs in enhanced capability of cancer cell invasion and the acquired resistances to chemotherapy of breast cancer. The potential therapeutic effects of all-trans retinoic acid (ATRA), which has been used in the management of certain hematologic malignancies and solid tumors, including breast cancer [24], on the induction of differentiation of enriched BCSCS, inhibition of aggressive growth and sensitization to chemotherapeutic agent in MCF/C6 cells. The results indicate that ATRA is a promising candidate to target radioresistant breast cancer cells with enrichment of BCSCs.

## Methods

### Reagents

ATRA was purchased from Sigma–Aldrich (St. Louis, MO), and was dissolved in dimethylsulphoxide (DMSO) as stock solution. Primary antibodies for Involucrin, Sydencan-3, and E-Cadherin were purchased from Santa Cruz Biotechnology (Santa Cruz, CA). Anti-CD44 antibody was from ABGENT (San Diego, CA). Anti-β-actin antibody was from Cell Signaling Technology (Beverly, MA). siRNA oligos for CD44 and control siRNA-A were also from Santa Cruz Biotech. Inc. Enzymes I-SceI was from New England Biolabs (Ipswich, MA).

### Cell culture

Human breast cancer MCF7 cells were from American Type Culture Collection (ATCC, Manassas, VA). Radiation-resistant MCF7/C6 cells were generated from MCF-7 cells by exposure to fractionized ionizing irradiation (FIR) with a total dose of 60 Gy of γ-irradiation (2 Gy per fraction, five times per week for 6 weeks) as previously described [17]. MCF7 and MCF7/C6 cells were maintained in ATCC-formulated RPMI-1640 medium supplemented with 10 % Fetal bovine serum (FBS), 5 % sodium pyruvate, 5 % nonessential amino acid, 100 U/mL of penicillin, and 100 mg/mL streptomycin in a 37 °C incubator (5 % CO_2_). To maintain the radiation-resistant phenotype, MCF7/C6 cells were also frequently exposed to irradiation (IR) at 2Gy for five times per week and radioresistance was validated before each designated experiment.

### Clonogenic survival assay

Cells in log-phase were plated and then immediately treated with indicated treatment. 24 h later, cells were washed twice with pre-warmed medium, and were then maintained in corresponding medium for 10–14 days and stained with crystal violet. Colonies consisting >50 cells were considered as survival colonies and directly scored using an inverted microscope. Average numbers for survival colonies were plotted versus untreated control to determine the survival fractions. When ATRA pretreatment applied, cells were treated 10 μM ATRA for 72 h. Cells were then re-plated and treated with indicated chemodrugs for 24 h, and maintained in corresponding medium for colony formations as described above.

### Assays for invasion, migration and wound healing

MCF7 and MCF7/C6 cells in log-phase were trypsinized, and 5 × 10^4^ cells in growth medium containing 1 % FBS were re-seeded in 1× BME (Trevigen, Gaithersburg, MD) coated 8.0-μm pore size cell culture inserts (for 24-well plate, Millipore, Danvers, MA). Complete growth medium containing 10 % FBS was placed outside the chambers, and cells were allowed to invade toward the attractant of full-serum medium. Chamber filter processing and visualization/quantitation of invasion were performed, as previously described [25]. Cells migrated to bottom chamber were also visualized/quantified for migration analysis.

For wound healing analysis, 5 × 10^4^ cells were grown in monolayers in triplicate in 24-well plates for 72 h. The confluent monolayer was then scraped with a sterile tip. The migration into the wounded monolayer was assessed by microscopy. When siRNA transfection applied, cells were transiently transfected with SiRNA-CD44 or SiRNA-Control-A, and then maintained in complete medium for 72 h until confluent monolayer formed for wound healing analysis, or re-seeded in BME-precoated cell culture inserts for invasion/migration assays.

### Flow cytometry analysis

After treatments, cells were detached by using stempro® accutase (Life Technologies, Grand Island, NY), and washed twice with PBS. Cells were then stained with PE-conjugated anti-Sox2, anti-Oct3/4, and anti-NANOG antibodies, or co-stained with PE-conjugated anti-CD24 and FITC-conjugated anti-CD44 antibodies (BD Biosciences, San Jose, CA). In the process for staining of Sox 2, Oct3/4 and Nanog, BD Perm/WashTM buffer was also used per manufacture’s instruction. PE- or FITC-positive cells were quantified by flow cytometric analysis on Flow Cytometer LSRII (BD Biosciences, San Jose, CA). Up to 5 × 10^4^ cells were counted during flow cytometry analysis. For cell cycle analysis, cells were collected and fixed with 75 % ethanol, stained with propidium iodide and analyzed by flow cytometry with 5 × 10^4^ events counting per run, as described previously [26]. The percentage of cells in the G_1_, S, and G_2_/M phases of the cell cycle were determined by using Flowjo software (Flowjo data analysis software, OR).

### Immunoblot assay

Cell lysates were prepared in RIPA buffer with mild sonication, and subjected to SDS-PAGE gel for immunoblot assays. β-actin was included to determine equivalent protein loading.

### in vivo end-joining assay

in vivo end-joining assay was based on the reactivation of linearized plasmid as previously reported [27]. Briefly, cells were treated with 10 μM of ATRA, or DMSO as control, for 72 h, 1 × 10^5^ cells were then co-transfected with 1.2 μg linearized EJ5-GFP substrates (linearized with I-SceI) and 0.5 μg circular pDsReD-Express2-N1 (as transfection control) by using electroporation (Gene Pulse Xcell, Bio-Rad, Hercules, CA). After transfection, cells were plated and cultured in fresh complete medium for 72 h. In ATRA experiment, 10 μM ATRA was added into culture medium after transfections and DMSO was included as control. Flow cytometry analysis was performed with Fortessa Flow Cytometer (Fluofarma, Princeton, NJ). Up to 5 × 10^4^ cells were counted. The ratio of GFP-positive cells to DsRed-positive cells was used as a measure of end-joining efficiencies.

### Tumor initiating test

Tumor initiating test was conducted following the described methods [19, 28] and the protocol was reviewed and approved by the Chancellor’s Animal Research Committee (ARC) at the University of California Los Angeles (ARC #2009–063–13). Six weeks old female NOD/SCID mice (Jackson Lab, Bar Harbor, ME) were pretreated for 5 days with estrogen pellets (Innovative Research of America, Sarasota FL) and freshly prepared MCF7 and MCF7/C6 cells were resuspended in serum-free PBS/Matrigel mixture (1:1 V/V), and 1×103 cells were inoculated subcutaneously to bilateral franks of same animal. Tumorigenesis was assessed twice a week with palpation. Tumor sizes were determined from caliper measurements of tumor length (L) and width (W) according to the formula (LxW2)/2.

### Statistical analyses

Statistical analyses were performed using the Student’s t-test. A *p* value <0.05 was considered as significant (*).

## Results

### Enhanced cancer cell invasiveness and migration of radiation-resistant MCF7/C6 cells

**Fig. 1:**
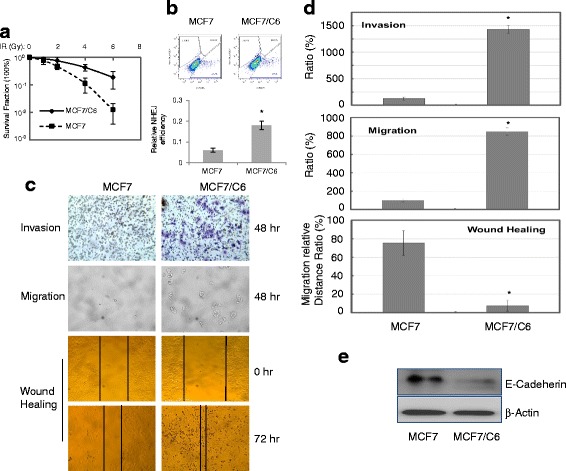
Radiation-resistant MCF7/C6 cells are more invasive cancer cells. **a** Increased radioresistance measured by clonogenic survivals of MCF7 and MCF7/C6 cells. **b** NHEJ efficiency measured by in vivo EJ assay. Cells were co-transfected with linearized EJ5-GFP plasmid and control pDsRed, and were then treated with 2 Gy of IR. Re-circulated EJ5-GFP was counted by flow cytometry analysis 72 h after transfection. **c** Representative images for transwell invasion assay and wound-healing assays (top: invasion assay; middle: migration assay; bottom: wound healing assay). **d** Relative quantitation of cellular invasiveness, migration and wound healing ability in MCF7/C6 cells compared with the wild type MCF7 cells. **e** Western blots of E-Cadeherin in MCF7 and MCF7/C6 cells. β-actin was included for equivalent protein loading. Data represent the average from at least three independent experiments. *Indicates statistical significance (*p* < 0.05)

Radiation in cancer treatment is intended to destroy cancer cells by damaging their DNA, and the resistance of cells to IR is thus modulated by three intimately related cellular processes, including DNA damage repair [29]. In this study, we first verified the radioresistance of MCF7/C6 cells. We found that the clonogenic survival rate was enhanced in MCF7/C6 cells to about 12-fold when compared to that of wild type MCF7 cells (Fig. 1a). Using in vivo end-joining assay, we detected the DNA repair capacity in MCF7/C6 versus wild type MCF7 cells and the results showed that NHEJ (non-homologous end-joining) DNA repair efficiency was about two-folds in MCF7/C6 cells compared to the wild type MCF7 cells (Fig. 1b). In agreement with NHEJ being an indicator of intrinsic DNA damage repair capacity [29, 30], these results indicate that DNA repair cacapicity plays a role in signaling the radioresistant phenotype of MCF7/C6 cells.

It has been previously shown that HER2-positive cells in MCF7/C6 were with increased invasiveness [19]. In an attempt to test whether MCF7/C6 cells have overall changes in cancer cell invasiveness and migration, we performed the assays in MCF7 and MCF7/C6 cells. We observed that the capabilities of cancer cell invasion/migration were dramatically enhanced in MCF7/C6 cells versus parental MCF7 cells. MCF7/C6 cells also showed increased ability for wound healing (Fig. 1c, d). In addition, a substantial amount of E-cadherin, a protein prominently associated with tumor invasiveness and metastatic dissemination [31], was found to be reduced in the MCF7/C6 cells (Fig. 1e).

### Enrichment of stem cell-like cancer cells in MCF7/C6 cells

**Fig. 2:**
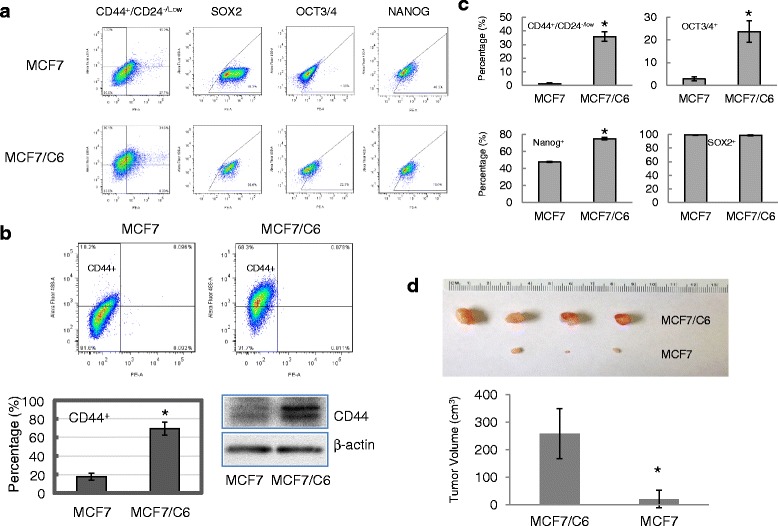
Enrichment of BCSCs in MCF7/C6 cells. **a** Flow cytometry analysis for different stem-cell surface markers in MCF7 and MCF7/C6 cells (*left*); **b** Increased CD44 expression in MCF7/C6 cells comparing to parental MCF7 cells. *Top*: flow cytometry analysis of CD44 expression; *Bottom left*: diagram showing the percentages of cell populations with CD44 expression; *Bottom right*: Western blot analysis for CD44 protein expression. Data represent the average from at least three independent experiments. **c** Diagram (*right*) showing the changes of the cell fractions with corresponding positive stem cell markers. **d** Tumorogenesis of MCF7/C6 cells verses MCF7 cells. Top: images for collected tumors from Tumor initiating test; *bottom*: diagram showing the average of tumor volumes. *Indicates statistical significance (*p* < 0.05)

We next examined the potential enrichment of stem cell-like cancer cells, or cancer stem cells (CSCs), in MCF7/C6 cells. Our previous study has revealed the enrichment of HER2^+^/CD44^+^/CD24^-/low^ cancer stem cell population in MCF7/C6 cells. In this study, we used cancer stem cell surface marker CD44^+^/CD24^-/low^, a first described marker for BCSCs [32, 33], and embryonic stem cell markers Oct3/4 [34], Sox II [35] and Nanog [36] to determine the putative cancer stem cells. Flow cytometry analyses showed significant increases of cell populations with positive staining of CD44^+^/CD24^-/low^ (from 1.26 ± 0.52 to 35.8 ± 3.41), Oct3/4 (2.78 ± 0.87 to 23.7 ± 4.66) and Nanog (from 47.6 ± 2.33 to 74.1 ± 4.27) in MCF7/C6 cells (Fig. 2a, c). In addition, we also detected increase of CD44-positive population, a determinant cell membrane protein in cell migration and invasion [37], in MCF7/C6 cells, which was further confirmed by western blot analysis (Fig. 2b, c). In NOD/SCID mouse, we found that all the sites inoculated with MCF7/C6 cells (1000 cells/injection) developed tumors (4/4) with an average volume of 259 mm^3^ at day 35; whereas three of four sites inoculated with the same number of MCF7 cells showed detectable tumors with an average volume of 20 mm^3^ (Fig. 2d and Additional file 1: Figure S1). MCF7/C6 cells also showed shorter latency for forming tumors when compared to MCF7 cells (16 ± 5 days versus 26 ± 2 days). Thus, the results of tumor initiating test suggested that radioresistant MCF7/C6 cells are more tumorigenic than parental MCF7 cells.

### Knocking-down CD44 expression inhibited the aggressive growth of MCF7/FIR C6 cells

**Fig. 3:**
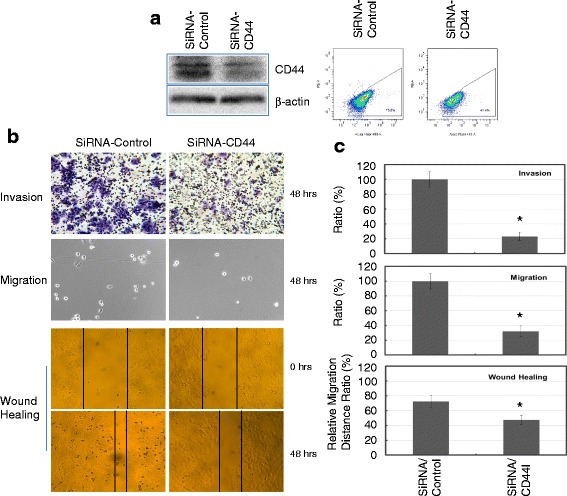
CD44 inhibition reduced invasiveness, migration and the ability of wound healing in MCF7/C6 cells. **a** siRNA transfection knocks down CD44 expression in MCF7/C6 cells. *Left*: Western blot showing the inhibition of CD44 in MCF7/C6 cells after transfection of siRNA-CD44 for 48 h; *Right*: Flow cytometry analysis showing the decrease of cell fractions with CD44-positive expression in MCF7/C6 cells with transfection of siRNA-CD44. **b** Representative images for cancer cell invasion, migration and wound-healing assays in MCF7/C6 cells with or without CD44 inhibition. **c** Quantitation of invasiveness, migration and wound healing ability in MCF7/C6 cells with CD44 inhibition. Data represent the average from at least three independent experiments. *Indicates statistical significance (*p* < 0.05)

Members of the CD44 family of transmembrane glycoproteins, in particularly CD44v6 isoforms, were shown to be metastatic determinants of tumor cells, and the expression of several CD44 proteins correlates with aggressive stages of various human cancers. Thus, CD44 has been considered as a therapeutic target for metastasizing tumors [38–40]. In CD44-overexpressed MCF7/C6 cells, siRNA-mediated CD44 inhibition led to a reduction in cell invasiveness and migration by near 70 % (Fig. 3). The gap filling rates were also reduced (near 8-folds) by knocking-down of CD44 in MCF7/C6 cells. These results indicate that CD44-expressing BCSCs are indeed enriched in the radioresistant MCF7/C6 population, and CD44 can be used as an effective therapeutic target to treat radioresistant breast cancer.

### ATRA induces differentiation and inhibits cancer cell invasion in MCF-7/C6 cells

**Fig. 4:**
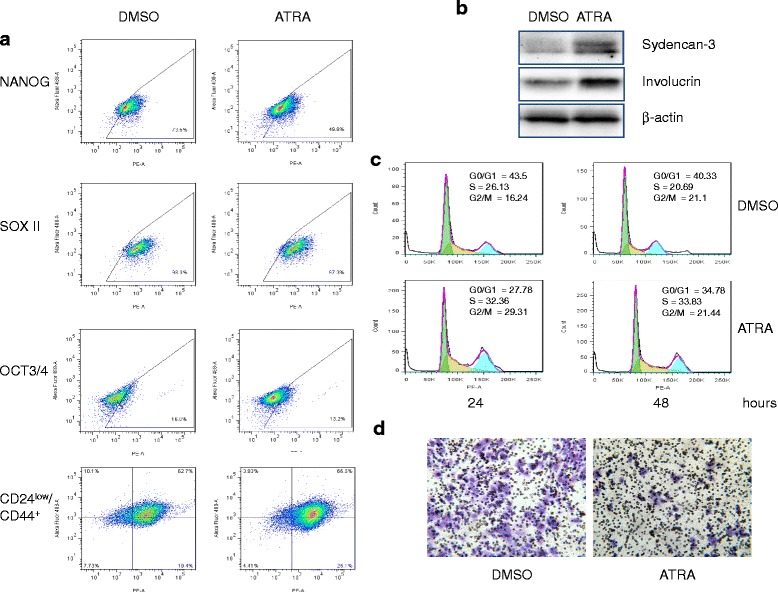
ATRA induces differentiation of MCF7/C6 cells. **a** Flow cytometric results for stem-cell surface markers in ATRA-treated MCF7/C6 cells. MCF7/C6 cells were treated with 1.0 μM of ATRA for 72 h, and were then analyzed by flow cytometry assay. **b** Westen blots showing that treatment with 1.0 μM ATRA for 72 h induces expressions of differentiation marker involucrin and Syndecan 3 proteins in MCF7/C6 cells. β-actin was included for equivalent protein loading. **c** The effect of ATRA on cell cycle progress in MCF7/C6 cells. Cells were treated with 1.0 μM ATRA, and then analyzed by flow cytometry. **d** Representative images showing ATRA treatment reduces the invasiveness of MCF7/C6 cells. Cells were pretreated with 1.0 μM of ATRA for 72 h, and invasion assay was performed as described in Materials

ATRA is routinely used as therapeutic agent to induce differentiation of leukemic stem cells in acute promyelocytic leukemia [41]. ATRA has also been reported to induce differentiation in cancer stem cells, including BCSCs [42–44]. Given the evidences above showing that radioresistant MCF7/C6 cells are with enrichment of CSCs, we thus tested the potential effects of ATRA on differentiation of MCF7/C6 population. Our results showed that treatment with 10 μM of ATRA for 72 h significantly reduced the percentages of cell fractions of CD44^+^/CD24^-/low^-positive (from 28.1 ± 2.38 to 4.27 ± 0.51) and NANOG-positive (from 72.8 ± 4.88 to 50.2 ± 3.79), and slightly reduced the percentage of cells that were OCT3/4-positive (from 16.6 ± 1.52 to 12.9 ± 2.33). Exposure to ATRA also increased the expressions of differentiation markers, involucrin and syndecan 3 [45, 46], in MCF7/C6 cells (Fig. 4a and b, and Additional file 1: Figure S1B). In addition, cell cycle analysis showed that ATRA treatment caused increases of S phase in MCF7/C6 cells after exposure for 24 h when compared to that of untreated control (from percentage of 21.53 ± 1.63 to 31.26 ± 1.82) or to that of DMSO treatment (from percentage of 25.88 ± 2.14 to 31.26 ± 1.82), which support the concept that that ATRA could induce cell proliferations in quiescent BCSCs population [47]. As expected, we also found that ATRA treatment reduced the percentages of invasive cells in MCF-7/C6 cells (Fig. 4d and Additional file 1: Figure S1C).

### ATRA enhances sensitivity of MCF7/C6 FIR cells to radiation treatment

**Fig. 5:**
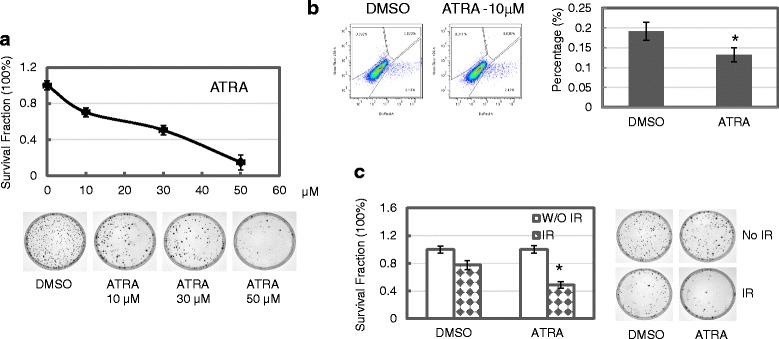
ATRA exposure increases the sensitivities of MCF7/C6 cells to radiation treatment. **a** Clonogenic survival of MCF7/C6 cells exposed to ATRA treatment. Cells were plated and treated with indicated doses of ATRA for 24 h, and cells were then cultured for colony formation. **b** ATRA exposure reduces NHEJ activity in MCF 7 FIR cells, and increases radiosensitivity. Cells were co-transfected with control pDsRed and linearized EJ5-GFP plasmid, and were then treated with 1.0 μM of ATRA for 72 h. in vivo EJ5 activity was measured as described in Materials. Left: in vivo EJ5 assay; Right: diagram showing the inhibition of EJ5 reunion ability in ATRA-treated cells; **c** Clonogenic survival assay was performed to determine the changes of sensitivity in cells treated with 2Gy ionizing radiation. Cells were pretreated with 1.0 μM of ATRA, or DMSO as control, for 72 h, and 500 cells were then plated and irradiated with 2 Gy of IR. *Left*: ATRA treatment reduced clonogenic survival of irradiated cells. *Right*: demonstrative images for colony survival of irradiated cells. Data represent the average from at least three independent experiments. *Indicates statistical significance (*p* < 0.05)

To further evaluate the potential therapeutic impacts of ATRA on breast cancers cells with acquired radiation resistance, we tested whether ATRA treatment could change the sensitivities of MCF7/C6 cells to radiation and chemotherapeutic treatments. For this, we first examined the direct cytotoxic effect of ATRA on MCF7/C6 cells and results indicate that treatment with ATRA at the concentration ranging up to 5 μM induced dose-dependent inhibition on clonogenic survival (Fig. 5a). We next examined the effects of ATRA on cellular capability of DNA damage repair and radiation sensitivity in MCF7/C6 cells. As shown in Fig. 5b, treatment with 10 μM of ATRA for three days reduced end-joining activity with statistical significance (from 0.192 ± 0.023 to 0.132 ± 0.018, *p* = 0.0352). In addition, pretreatment with ATRA at 10 μM for 72 h sensitized MCF7/C6 cells to radiation treatment, as determined with clonogenic survival (from percentage of 77.41 ± 5.30 to 48.54 ± 4.83, *p* = 0.0162, (Fig. 5b and c).

### ATRA enhances sensitivity of MCF7/C6 cells to chemotherapy

**Fig. 6:**
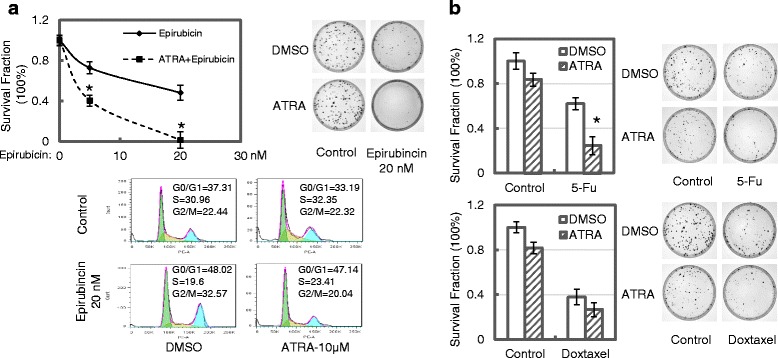
ATRA exposure increases the sensitivities of MCF7/C6 cells to chemotherapeutic treatments. **a** Effect of ATRA on clonogenic survival and cell cycle distribution in epirubincin-treated MCF7/C6 cells. Cells were pretreated with 1.0 μM ATRA, or DMSO as control, for 72 h, and 500 cells were then plated and treated with indicated concentrations of Epirubicin. 24 h later, cells were washed with fresh medium and were then maintained for colony formation assay, or collected for cell cycle analysis. *Top left*: Survival curve for colony formation; *Top right*: demonstrative images for colony survival of epirubincin-treated cells. Bottom: ATRA-induced cell cycle changes in epirubincin-treated cells. **b** Effect of ATRA on responses of MCF7/C6 cells to treatments of 5-Fu and Doxetaxel. Cells were pretreated with ATRA as described above, and were then treated with 1.0 μg/mL of 5-Fu or 0.5 nM of Doxetaxel for 24 h. Colony formation experiments were then performed. *Top left*: Diagram showing the change of colony formation in cells exposed to 5-Fu treatment; top right: demonstrative images for colony survival of 5-Fu-treated cells; Bottom left: Diagram showing the change of colony formation in cells exposed to Doxetaxel treatment; Bottom right: demonstrative images for colony survival of 5-Fu-treated cells. Data represent the average from at least three independent experiments. *Indicates statistical significance (*p* < 0.05)

With clonogenic assays, we also observed that pretreatment with ATRA enhanced clonogenic cell killing effects of epirubincin and 5-Fu on MCF7/C6 cells. However, it did not affect the clonogenic survival of cells treated with 1 nM of Doxetaxel (Fig. 6). We further noticed decrease of G_2_/M distribution of cells in epirubincin-treated MCF7/C6 cells when cells were pretreated with ATRA, suggesting that the ATRA pretreatment-enhanced cell killing effect of epirubincin in cells may occur in G_2_/M phase of cell cycle.

## Discussion

Radioresistance of cancer cells may arise from self-repair mechanisms (mainly DNA damage repair) or repopulation of radioresistant cancer stem cells, or both [48]. Data presented here indicate that the radioresistant MCF7/C6 population derived from long-term fractionated doses of radiation is with enrichment of BCSCs and enhanced capability of NHEJ repair. Compared to parental MCF7 cells, MCF7/C6 cells are aggressive with increased capacity of invasiveness and migration, and inhibition of CD44 expression could effectively reduce cancer cell invasiveness and migration in MCF7/C6 cells. Most important, our data demonstrated that treatment with ATRA can induce differentiation of the enriched BCSCs in MCF7/C6 cell population and sensitized them to chemotherapeutic agent epirubincin.

More than 60 % cancer patients worldwide use radiotherapy for the control of tumor growth during the course of their disease. However, in spite of significant advancements in tumor imaging and precise of tumor dose calculation and delivery, the rate of total tumor growth control by radiotherapy remains disappointing. Although radiation therapy can decrease the risk of local cancer recurrence and improves survival, clinical evidence has shown the detrimental effect of treatment interruptions on tumor control in breast cancer patients [49]. Interestingly, radiation can also induce a BCSC phenotype in differentiated breast cancer cells [21, 22], and CSCs-mediated tumor innate resistance to cytotoxic agents thus become major clinical challenges towards the complete eradication of minimal residual disease in cancer patients [50]. CSCs are also likely to play essential roles in the metastatic spread of primary tumors because of their self-renewal capability and their potential to give rise to differentiated progenies that can adapt to different target organ microenvironments [51–54]. Preclinical study has suggested differentiation therapy to be one of the promising strategies for targeting BCSCs in breast cancer [55]. Thus, targeting these enriched putative BCSCs in breast cancer cells after sublethal doses of radiation treatment may have important clinical impact for breast cancer patients. To this setting, radioresistant MCF7/C6 used in this study is a useful experimental model to mimic the radioresistant lesions in the clinic, especially for the therapy-resistant phenotype of metastatic tumors. MCF7/C6 cells were derived from MCF7 cells after fractionized ionizing radiation and are with developed radiation resistance [16, 19, 56]. Characterization and elucidation of the mechanistic insights and potential therapeutic target to this unique radioresistant, BCSCs-enriched population which is highly relevant to the clinic recurrent/metastatic lesions, will generate informative data for the benefit of breast cancer patients. Our present work demonstrates the increases of putative CSCs populations in MCF7/C6 cells. Compared to parental MCF7 cells, MCF7/C6 cells also exhibited enhanced capabilities for cancer cell invasion and migration, indicating increased potential for metastasis. Thus, radioresistant MCF7/C6 with BCSCs enrichment is a useful experimental model to mimic the radioresistant lesions in the clinic, especially for the therapy-resistant phenotype of metastatic tumors. Preclinical study has suggested differentiation therapy to be one of the promising strategies for targeting BCSCs in breast cancer [55]. Our data also showed that inhibition of CD44 expression could effectively reduce cancer cell invasiveness and migration in MCF7/C6 cells (Fig. 3).

In this study, we demonstrated the potential therapeutic effects of ATRA on MCF7/C6 cells. Retinoids and its derivatives such as ATRA are promising anti-neoplastic agents endowed with both therapeutic and chemo-preventive potential because they are able to regulate cell growth, differentiation and apoptosis [57–59]. We previously have showed the inhibitory effects of ATRA on proliferation and cancer cell migration of breast cancer cells [60]. ATRA has also been recently demonstrated of the ability to induce cancer stem cell differentiation [42]. We showed here that ATRA can induce differentiation of enriched BCSCs in MCF7/C6 cells, and inhibit cancer cell invasiveness/migration and increase the sensitivities of cells to radiation treatment and to the treatments of epirubincin and 5-Fu of this cell population. These results thus not only indicate potential clinic impacts of differentiation treatment with ATRA as single agent for BCSCs in therapy-resistant breast cancers, but also suggest approaches with combination of ATRA and epirubincin, or other standard-anti-breast cancer chemotherapy, as novel therapeutic strategy for clinic management aiming to minimize the risk of recurrent/metastasis, the major life-threatening tumors in many cancer patients [61].

## Conclusions

Our study suggests a potential clinic impact of ATRA as a chemotherapeutic agent for treatment of radiation-resistant breast cancer. The study also provides a rationale for ATRA as a sensitizer of Epirubincin, a first-line treatment option for breast cancer patients.

## Availability of data and materials

Not applicable.

## Additional file

Additional file 1: Figure S1. A. Representative images for H.E staining of the tumors formed in NOD/SCID mouse in tumor initiative test. B. Diagrams showing the effects of ATRA on percentages of cell populations with positive staining of stem cell markers Nanog, OCT3/4 and CD44^+^/CD24^-/low^ in MCF7/C6 cells. C. Diagrams showing the ratios of cancer cell invasiveness MCF7/C6 cells treated with ATRA as shown in Fig. 4d. Data represent the average from at least three independent experiments. *Indicates statistical significance (*p* < 0.05). (PDF 159 kb)
