# Supplementary figures and images for: All-trans retinoic acids induce differentiation and sensitize a radioresistant breast cancer cells to chemotherapy
Source: BMC Complement Altern Med. 2016 Mar 31;16:113. doi: 10.1186/s12906-016-1088-y (PMC4815257; doi:10.1186/s12906-016-1088-y)

Supplemental figure S1

A

MCF7/C6

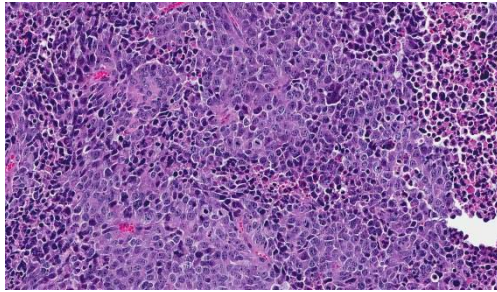

MCF7

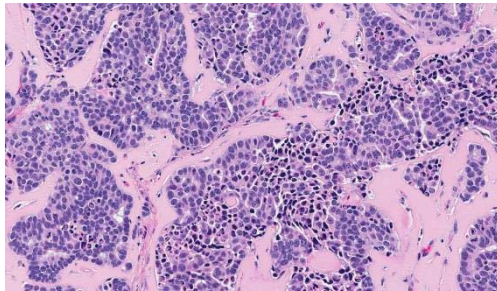

C

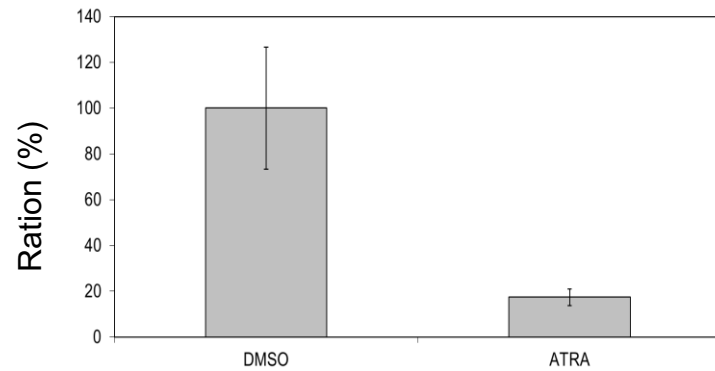

B

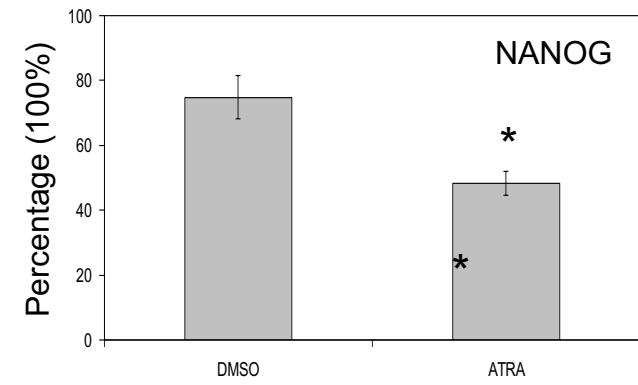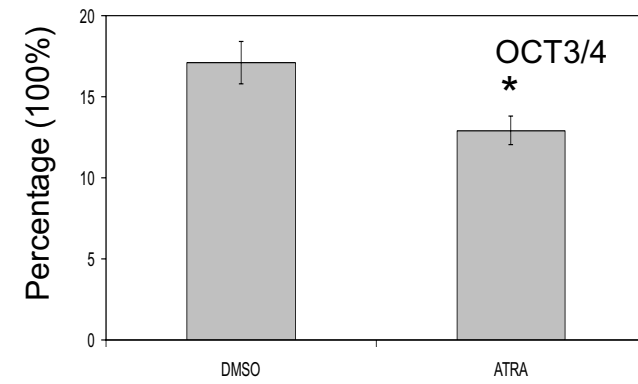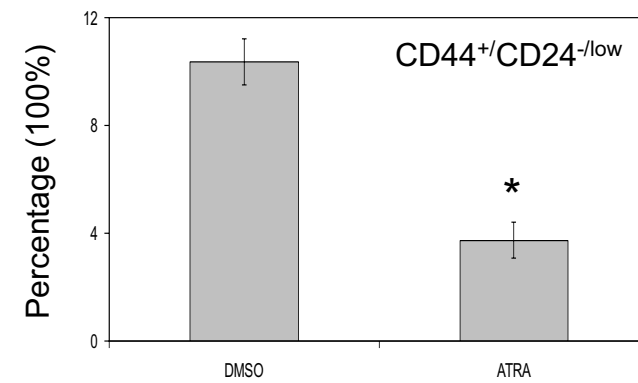

Supplement: Additional file 1: Figure S1. — A. Representative images for H.E staining of the tumors formed in NOD/SCID mouse in tumor initiative test. B. Diagrams showing the effects of ATRA on percentages of cell populations with positive staining of stem cell markers Nanog, OCT3/4 and CD44+/CD24-/low in MCF7/C6 cells. C. Diagrams showing the ratios of cancer cell invasiveness MCF7/C6 cells treated with ATRA as shown in Fig. 4d. Data represent the average from at least three independent experiments. *Indicates statistical significance (p < 0.05). (PDF 159 kb) [file 12906_2016_1088_MOESM1_ESM.pdf]
